# Improving Network-Based Anomaly Detection in Smart Home Environment

**DOI:** 10.3390/s22155626

**Published:** 2022-07-27

**Authors:** Xiaonan Li, Hossein Ghodosi, Chao Chen, Mangalam Sankupellay, Ickjai Lee

**Affiliations:** Discipline of Information Technology, College of Science & Engineering, James Cook University, Townsville, QLD 4811, Australia; xiaonan.li@my.jcu.edu.au (X.L.); chao.chen@jcu.edu.au (C.C.); mangalam.sankupellay@jcu.edu.au (M.S.); ickjai.lee@jcu.edu.au (I.L.)

**Keywords:** smart home security, anomaly detection, mechanical learning

## Abstract

The Smart Home (SH) has become an appealing target of cyberattacks. Due to the limitation of hardware resources and the various operating systems (OS) of current SH devices, existing security features cannot protect such an environment. Generally, the traffic patterns of an SH IoT device under attack often changes in the Home Area Network (HAN). Therefore, a Network-Based Intrusion Detection System (NIDS) logically becomes the forefront security solution for the SH. In this paper, we propose a novel method to assist classification machine learning algorithms generate an anomaly-based NIDS detection model, hence, detecting the abnormal SH IoT device network behaviour. Three network-based attacks were used to evaluate our NIDS solution in a simulated SH test-bed environment. The detection model generated by traditional and ensemble classification Mechanical Learning (ML) methods shows outstanding overall performance. The accuracy of all detection models is over 98.8%.

## 1. Introduction and Background

Smart Home (SH) is the implementation of Internet of Things (IoT) devices in a home environment. SH appliances are essentially resource-constrained network devices, and users can execute predefined automation tasks remotely on these devices. IoT Analytics [1] estimate that 14.4 billion connected IoT devices are active worldwide at the end of 2022 and forecasts that the number will increase to 27.1 billion at the end of 2025. Telsyte [2] indicates that 6.3 million Australian households have at least one SH product at the end of 2021. The average number of IoT devices in Australian homes was 20.5 in 2021, which will increase to 33.8 by 2025. The massive market demand urges manufacturers to develop SH products with new functionalities as quickly and cost effectively as possible to compete with others and attract new users. As a result, the security of the product has a lower priority than its functionality, and, it is often overlooked. Various studies have revealed that commercial SH devices along with their corresponding software have vulnerabilities [3,4,5,6] that lead to critical security threats to authorization, authentication, key management and access control [7,8]. Vulnerabilities such as default/weak login, unclosed telnet/SSH port, backdoor and permission over-privilege commonly exist in most commercial SH products, which can be easily exploited to launch an attack [9]. The number of captured attacks targeting SH devices has increased dramatically in the past few years. In 2016, 10,263 different SH devices were remotely controlled to host Mriai [10]. Those botnets are being used to launch a large-scale DDOS attack to disrupt the services of Krebsonsercurity.com and Dyn. After the release of Mriai source code, several variants have been identified in a short time, e.g., Hajime and Satiro. Compared to the IoT-based attack with a traditional cyberattack, the damage of IoT-based attacks increased simultaneously with the increasing deployment of insecure IoT devices. Based on the functionality of the targeted SH devices, the intruders are able to gain access not only to the residents’ private information but also potentially to enter the physical residential environment. The security issues within an SH can be described from multiple perspectives. IoT device attacks can be categorized based on the Traditional ITU-T Architecture (e.g., physical, network, application and protocols for data encryption) or from a user-provider perspective [11] (e.g., user layer, service layer, virtualization layer and physical layer). However, the essence of SH attacks is to exploit the target devices’ vulnerabilities. These vulnerabilities may exist in end point IoT devices, cloud services and communication protocols. An intrusion of an SH can be described as an unauthorized user gaining access to the resources of an IoT device within HAN via different attack vectors (existing vulnerabilities within the device). SH users generally lack security awareness [12] (e.g., using default or weak passwords, not frequently updating or installing security patches). Furthermore, due to the hardware resources limitation of current IoT devices, the implemented defence techniques suffer from balancing the trade-off between security, cost and performance.Therefore, identifying attacks in an SH at the initial phase is significant to general SH users, and it is necessary to design and develop a security solution specifically for the protection of IoT implementations in the case of an SH.

A Network-Based Intrusion Detection System (NIDS) is designed to detect abnormal events by analysing HAN traffic. Currently, there are two types of NIDSs that exist, which are signature-based NIDSs (S-NIDSs) and anomaly-based NIDSs (A-NIDSa). An intrusion can be detected if the monitored HAN traffic breaks the predefined rules or matches with the known attack signature. Commercial products prefer misuse detection [13] due to efficiency and accuracy, e.g., Snort [14]. However, S-NIDSs are unable to detect the unknown attack and require constant updates of the predefined rules or new attack signatures. Hence, S-NIDSs are not suitable to be implemented in SH. A-NIDSs attempt to quantify the characteristics of acceptable network behaviours of IoT devices to establish a normal profile in HAN. An A-NIDS compares the monitored traffic with the normal profile; the observed deviation will be considered as an intrusion. An A-NIDS [15] is more suitable to be implemented in SH IoT devices due to: (1) less memory requirement and little maintenance after system installation; and (2) the ability to detect unknown attacks. Three modelling techniques are currently being widely used in A-NIDSs to establish the normal HAN profile [16]: statistic model-based A-NIDS, Machine-Learning-based (ML) A-NIDS and knowledge-based A-NIDS. Both statistic model-based A-NIDS and knowledge-based A-NIDS require the user to have a solid knowledge background of network security to form the normal profile of the SH appliance. Moreover, the detection result highly depends on the appropriately selected traffic event of SH appliance activities. An ML-based A-NIDS is suitable for the general SH user; however, some drawbacks need to be addressed before implementation, such as: (1) lack of training data, commonly used benchmark datasets are outdated and cannot represent real HAN traffic, e.g., KDDCUP99 [17] and NSL-KDD [18]; and (2) generally, an HAN profile generated by different ML algorithms is computational costly (high time and space complexity), especially when dealing with large-scale datasets that have high-dimensional properties or nonlinear feature spaces [19].

To address the issues described above, we present User-Command-Chain (UCC) as a novel method to assist ML-based classification algorithms for generating anomaly detection models in an HAN. The essentials of anomaly detection in an HAN are based on the facts: a command received by an SH device is different between a legitimate user and an attacker in three aspects: time, location and payload. The generated response traffic, therefore, will be different. An End-SH-IoT-Device (EID) executing a predefined task requires receiving the specific command from the correlated Control Device (CD). In some cases, an SH control platform may involve forwarding such a command to the EID. From our observation, the communication among SH devices shares some common characteristics: small payloads, stable packet length and data exchange entities are normally fixed. Moreover, if the command is the same, despite the false packets during the information exchange, the network behaviour pattern is certain. The main aim of this study is to investigate intrusion in the initial phase of SH environments. We conduct this study with a simple SH environment. Our detection model identifies the network anomalies based on users’ commands (used to interact with specific device functions) by observing the responding network behaviours of triggered functions in the HAN. Our work first generates a benign model by observing the communication that delivers one particular command from a CD to an EID within a specific time slot, and then uses other deployed EID network behaviours as support evidence to represent the current SH condition and enhance the detection. The contributions of this study are listed as follows:

1. We propose a new method to pre-process the network packets data for training an ML-based A-NIDS detection model in an HAN environment. UCC has proposed to: (1) establish a good understanding of triggered SH IoT device network behaviours based on the users’ command, and (2) handle imbalance and high-volume data in the captured HAN traffic datasets;
2. We have set up a test-bed in a home environment to simulate the actual usage of an SH. We collected traffic data from our experimental test-bed instead of outdated simulation-based datasets;
3. Three types of network attacks have been used to evaluate the detection method in a test-bed environment. The result indicates UCC has improved both the accuracy and efficiency of A-NIDS detection.

The rest of the paper is structured as follow. In Section 2, we will present state-of-the-art A-NIDS related to our works. We higlight the SH threats and issues within the current A-NIDS and propose our solution in Section 3. Section 4 evaluates the performance of the proposed solution. And finally, the conclusion and future works will be presented in Section 5.

## 2. Related Works

**Network Intrusion Detection Systems (NIDS)** have been deployed at strategic points in the network infrastructure, such as the switch spanning port, network tap (terminal access point), gateway and router [20]. To detect attacks, the NIDS captures and analyses the stream of inbound and outgoing packets in real-time. In the scenario where the user interacts with an SH EID, the normal user behaviour in a certain period of time is regular. Consequently, legitimate residents’ activity patterns, based on their daily interactions with all deployed SH devices, can be used as a reference for generating SH security policies and used for detecting abnormal events within a certain period of time. The corresponding network behaviour of an EID can be considered as network signatures of the IoT device. Apthorpe et al. [21] use traffic fingerprint (traffic shape-based device network signature) to infer SH devices’ activities. Typically, an SH device only communicates with manufacturer-operated servers based on the assigned tasks; therefore, only a few packets are required to identify specific activity. PingPong [22] and HomeSnitch [23] use network flow data to establish a detailed signature based on the event inference. In PingPong, a state machine has been used to maintain packets’ sequence of the EID event signature. Once the monitored packets do not match with the predicted packets in modelled sequence, the abnormal event is detected, and the following packets will then be ignored. In HomeSnitch, Random Forest, K-nearest-Neighbors and Gradient Boost have been used to establish a normal network profile of a target EID; any deviation from the normal profile will be considered as the target EID being under attack.

**Machine-Learning-Based Network Intrusion Detection Systems** ML algorithms have been extensively applied in the field of NIDSs, especially classification algorithms such as Bayesian, Fuzzy Logic and support vector machine (SVM). The NIDS proposed by Puttini et al. [24] builds a behavioural model with posteriori Bayesian classification. This work assumes that different traffic profiles based on each event will influence the set of variables available for monitoring. The main disadvantages of the Bayesian classification-based IDS are: (1) detection results are highly dependent on assumptions about the behaviour events of the target system so that a deviation hypothesis may lead to detection errors, and (2) the dimensional and computational complexity of Bayesian classification IDS will increase exponentially with the increase in attributes. The NIDS proposed by Dickerson et al. [25] uses simple network traffic metrics combined with fuzzy rules to determine the likelihood of port scan attacks. The network activity is considered as normal if it lies within a given interval. The main disadvantages of fuzzy-logic-based IDS include: (1) high resource consumption, and (2) difficult to clearly define the criteria for attack detection; fuzzy rules are created by experts and may be time-consuming and labour intensive. Jayshree and Leena [26] have proposed an NIDS model based on an SVM and the best feature set selection algorithm with NSL-KDD datasets. The main disadvantage of an SVM-based IDS is the high communication overhead in the distributed environment owing to the need to send all time series of data from the end node to the analysis centre. Kou et al. [27] compared the performance of different machine learning methods on KDD CUP 99 datasets. The detect accuracy of the SVM outperforms Logistic Regression (LR), Naïve Bayes, Decision Tree (DT) and Classification and Regression Tree (CART).

**Class-Imbalanced Issue** Sampling, cost-sensitive learning, and one-class learning are the three main approaches to currently deal with the imbalanced classes issue in machine learning. Sampling includes over-sampling, under-sampling, and mixed sampling. Over-sampling is to generate multiple instances from a minority class such as SMOTE [28], ADASYN [29], and Borderline-SMOTE [30]. Under-sampling is to select some samples from the majority class such as Tomeklink [31], ENN [32] and NearMiss [33]. Mixed sampling refers to the combination of under-sampling and over-sampling. The aim of sampling is to balance the classes in datasets. The disadvantages of sampling include inefficiency, easy to be affected by noise, and unable to apply to datasets that cannot calculate the distance of each sample. Cost-sensitive learning assigns unequal cost to different classes, such as a higher cost to the minority class and a smaller cost to the majority class. Therefore, it reduces the classifier’s preference for the majority class. One-class learning is not to capture the differences between classes but focuses on model majority classes. Hence, it changes the detection problem from binary classification to a clustering issue, which identifies a test sample belonging to the majority class.

## 3. Anomaly Detection in Smart Home

### 3.1. Threat Model and Problem Description

Attack payload execution will negatively influence both the performance and status of the victim device, e.g., gaining unauthorised access to a service, resource or information. The traffic generated by such intrusion can be viewed as anomalies. Symptoms of the attack can be identified by inspecting the payload of network packets, e.g., DoS, probing attacks, User to Root (U2R) and Remote to Local (R2L). In this paper, we focus on identifying network anomalies in an HAN that are generated by attacks, which directly affect the network activities of an EID, or the sign of attack is visible in the HAN.

We assume SH devices contain default credentials, lack security features and have unpatched vulnerabilities. The attacker can compromise a deployed EID by directly connecting to the HAN or using the NAT hole-punching technique. Three types of network attacks have been selected to evaluate the detection model: port scan attack, SSH brute force attack and SYN flood attack.

Generally, there are four types of network attacks: DoS, probing attack, U2R and R2L [34]. The selected three attacks are widely adopted in current malware and attack scripts and play significant roles in the Cyber Kill Chain proposed by Lockheed Martin [35]. Port scan is a type of probe attack and is commonly used to identify the basic information of contained IoT devices (e.g., open port, carried OS and potential vulnerabilities). Brute force is a type of R2L attack and is commonly used to obtain login credentials. SYN flooding is a type of DoS attack; it can be used to induce the legitimate user to physically reboot the victim device to finish the malware installation process. As our study focuses on intrusion in the initial phase, the User to Root (U2R) attack which gains access to the local IoT device is not included in this study. This is one of the future directions of this study. In general, the attack packets account for a small proportion of the traffic in long-term network monitoring (e.g., 24 h). However, within the short-term network monitoring (e.g., 15 s), for the same IoT device, the number of traffic packets generated by attacks compared to executing predefined commands is much larger. All three chosen attacks generate a considerable number of abnormal traffic packets and cause imbalanced and large-volume issues in the collected datasets. In highly imbalanced classes of network attack datasets, the classifier always predicts the most common class, therefore leading to generating inaccurate models for detection.

### 3.2. End Device Behaviour and User-Command-Chain

SH IoT devices are generally resource-constrained and designed to perform a specific function with minimal physical device size. Hence, limited hardware features and software components are equipped in SH IoT devices. The predefined function of an EID restricts the tasks that an EID can perform. Therefore, the ways a user interacts with one deployed SH IoT device are also limited. Moreover, the predefined function feature of an SH IoT device will not be changed significantly in a short period of time. As a consequence, the traffic pattern of such an SH IoT device: (1) has fewer communication objects and a lower frequency of conversations based on predefined function features; (2) the transferred packets within the traffic between two SH appliances usually contain small-sized payload and unique packet length; and (3) low packet loss. Based on the above observations, we can conclude that the network behaviour of an activated EID function generated by the same user command has a similar traffic pattern, including connected devices/domains, packet sequences within the communication and individual packet length. Hence, it is possible to detect deviations from the normal profile when such Sh IoT devices are under attack.

We introduce UCC as a pre-processing method for an ML-based A-NIDS. UCC is a highly abstract statistic profile of one particular usage intention of an EID function within the specific time slot. A UCC is composed of three objects from the traffic unit generated by the triggered EID function: a source CD, a destination EID and a group of support evidence (the network behaviours of the rest of the deployed SH IoT devices). Collected packet data will split into groups based on the protocols of different layers of the TCP/IP model. Packets belonging to the transport layer will transform to flow data following the rule of IPFIX (IP Flow Information Export) [36]. Packets belonging to the application layer will count the frequency and be recorded in flow-like structure data. Entropy has been introduced to the UCC for representing a similar degree of all inbound/outbound flows/flow-like structure data within different UCC objects. We assume that the first time a new SH device is deployed in an SH: (1) this new EID will not contain any malware application; and (2) this new EID will not be selected as the attack target in a short period of time. Therefore, the traffic generated by triggering such an EID function can be considered as benign and is used for generating the normal profile. An in-progress IoT attack is detectable by identifying the deviation from the normal profile of a specific EID function. Although the detection cannot specify the types of attack, it can indicate the source of attack and target of the EID and achieve detection of the unknown attack.

### 3.3. Proposed Solution

**Figure 1:**
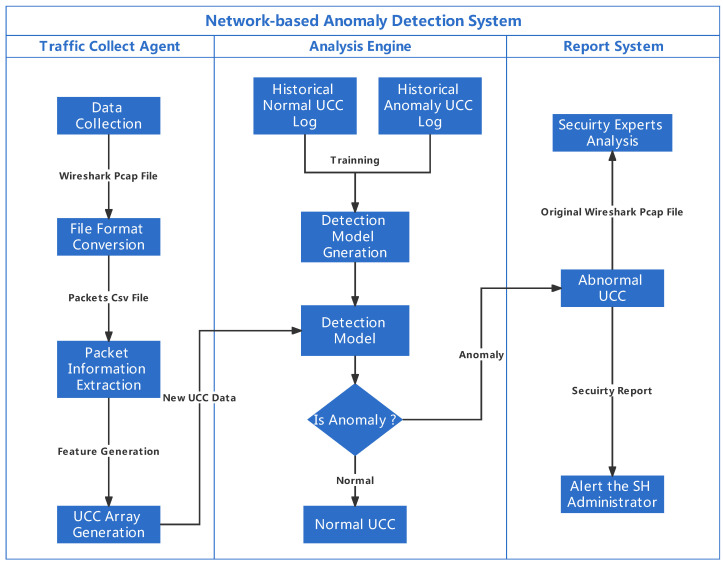
Network-based anomaly detection system.

The proposed ML-based A-NIDS has three main modules: a traffic collection agent, an analysis engine and a reporting system. The traffic collection agent deployed at the Home Gateway (HG) is responsible for collecting traffic data to generate the UCC based on the observation of the triggered EID function. The analysis engine has been implemented as a software application at a Raspberry Pi within the HAN. The UCC data generated in the traffic collection agent are used by the analysis engine as input of a classification-based ML algorithm to build the detection model. Furthermore, the analysis engine decides whether or not abnormal activities occur in the current time slot. After identifying the anomalies, the analysis engine will record the abnormal UCC in a log and forward the detection result to the report system. Based on the received detection result, the report system will: (1) alert the SH administrator of such an occurred security incident by email to take further actions in response; and (2) forward the abnormal data to security experts for further analysis. The overall detection model has been shown in Figure 1.

#### 3.3.1. Traffic Collection Agent

A traffic collection agent has been installed in HG to collect the inbound and outbound traffic data in real time. The traffic collection agent contains four main functions: data collection, file format conversion, packet information extraction and UCC generation. Wireshark and TCPdump have been used to collect traffic data and store it in a pcap format file. From the observation of our test-bed, the time interval of a process between one CD sending a command and an EID finishing the response to such a command is generally completed within 15 s. In our case, we collected the traffic data every 15 s from HG. Then the stored pcap file will be converted to csv format. Attributes that exist in the csv file to describe packets in the traffic unit include No., Time, Source, Destination, Protocol, Length and Info.

Once file format conversion is finished, we extract the key information from the packet of the CSV file. First, we split the data into three groups by two-round search: EID group, CD group and support evidence group. In the first-round search, we identify the triggered EID based on the existence of an activated function keyword in a packet. All inbound and outbound packets belonging to same EID will be categorised in a EID group. We identify the CD using backward trace in the second-round search. The process will terminate if the source IP equals one of the predefined control devices’ IP address. All inbound and outbound packets belonging to the same CD will be categorised in a CD group. The rest of the packets will be stored in the support evidence group based on the EID IP address. Based on the device group, the collected packets data are governed by:

(1) Device_Group,G={E,C,SE},

where *E* is the set of packets that belongs to the triggered end IoT device function; *C* is the set of packets that belong to the control device which triggered such EID function; and SE is the set of packets that indicate the current SH condition and can be used as support evidence to confirm such user interaction.

Second, within each group, collected packets data will transform to flow data or flow-like structure data based on the protocol attribute; for example, protocols belonging to the transport layer of TCP/IP model will transform to TCP flow based on five-tuple attributes ( Source IP, Source Port, Destination IP, Destination Port, and Protocol) and UDP flow based on three-tuple attributes (Source IP, Destination IP and Protocol). A three-tuple-like structure has been adopted to record the occurring frequency of packets using application layer protocols. To describe the network behaviour, for each flow and flow-like structure data, we extracted and recorded the packet information within the traffic unit, including total numbers of packets, the average length of the packet, the total length of packets and average time interval of send/receive packet. Moreover, specific string information within the Info attribute of the packet will be recorded to indicate the communication behaviour, e.g., 6 types of TCP Flags, 11 types of HTTP response status code, and 14 types of MQTT command messages.

Once packet information extraction is finished, based on the direction of communication, all collected flows and flow-like structure data belonging to the same device will be further split into four sub-groups: internal inbound, internal outbound, external inbound and external outbound. For each sub-group, three types of information will be collected: (a) entropy of group flows; (b) general group information that includes (1) number of flows, (2) number of packets to form flow, (3) average length of flows, (4) average length of packets to form flow, and (5) average time interval of send/receive of flows; and (c) key info within different types of flow based on protocols. The extracted sub-group information will be aggregated to form the UCC array, and the UCC array will send to the analysis engine to generate a detection model. The final UCC array can be represented as follows:

(2) $$\begin{array}{cc} UCC\_ & Array,U=\\ \; & \{\left\{E_{int\_in\_flow},E_{int\_out\_flow},E_{ext\_in\_flow},E_{ext\_out\_flow}\right\},\\ \; & \{C_{int\_in\_flow},C_{int\_out\_flow},C_{ext\_in\_flow},C_{ext\_out\_flow}\},\\ \; & \{SE_{int\_in\_flow},SE_{int\_out\_flow},SE_{ext\_in\_flow},SE_{ext\_out\_flow}\},\\ \; & \{Entropy\_Internal_{protocol\_1},Entropy\_External_{protocol\_1}\},\\ \; & \cdots \\ \; & \{Entropy\_Internal_{protocol\_n},Entropy\_External_{protocol\_n}\},\\ \; & \{Label\}\}. \end{array}$$

#### 3.3.2. Analysis Engine

The analysis engine contains two functions, which are detection model generation and anomaly detection. As we mentioned previously, we consider the newly deployed EID only performs benign activities. Therefore, historically collected UCC arrays in the log file are labelled as normal. We simulated attacks targeting such a device during user interact with a specific predefined function. The attack UCC arrays are generated from the collected attack scenario and labelled as abnormal. An analysis engine takes both normal and abnormal UCC arrays to train different ML algorithm classifiers and generate the detection model. In our study, the ML classification algorithms used to generate anomaly-based NIDS detection models include traditional classification methods (Logistic Regression, Naïve Bayes, Decision Tree, K-Nearest-Neighbors and Support Vector Machine) and ensemble classification methods (bagging-based method (Random Forest) and boosting-based method (XGboost)). One hot encoding technique has been adopted to convert categorical variables data to a form that could improve the prediction of each ML algorithm. Cross-validation and a hyperparameter tuning method have been applied to generate each model. Hyperparameter tuning is used to select the set of well-performing hyperparameters to configure each model; 10-fold cross-validation is used for avoiding the over-fitting issue.

During the detection, the currently received UCC array will be used as input of the detection model. The output predicts whether the current UCC array is classified as benign or anomalous. An ongoing attack will be detected if the A-NIDS model predicts the UCC array as an anomaly. Meanwhile, the abnormal UCC array along with its original pcap file will be sent to the report system for further processing.

## 4. Performance Evaluation

### 4.1. Test-Bed Smart-Home Environment and Scenarios

Smart-lamp and security camera have been selected in our simulated experiments for two reasons: (1) these two SH devices are most commonly deployed in a current SH, and the network activity patterns are profoundly different; (2) the network behaviours of these two SH devices can represent most of the current commercial SH appliances. The smart lamp can be viewed as a wireless switch; the predefined function (turn on/off) will only be triggered by the controller (e.g., a control hub) receiving the specific command. On the other hand, security cameras continuously upload the collected information to the SH control platform or an external server. The simulation scenarios include controlling the smart light on/off and monitoring the camera images externally: (1) turn the smart lamp on and off remotely; and (2) access the security camera video via an SH control platform and simultaneously turn the smart light on/off.

**Figure 2:**
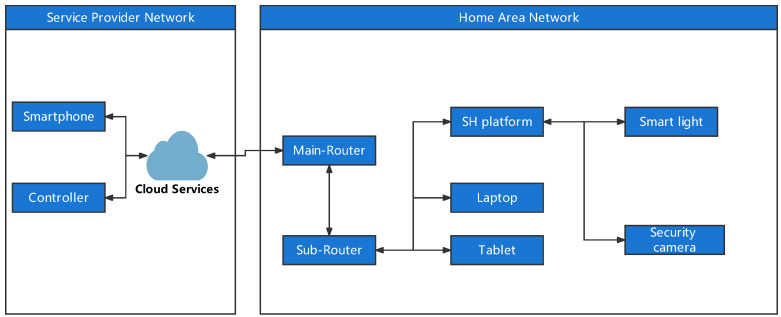
The architecture of test-bed SH.

In our experiment, all deployed devices are connected with each other via a Wi-Fi network (IEEE 802.11ac) with limited communication protocols, such as TCP, HTTP and MQTT; Frp NAT penetration technique (https://github.com/fatedier/frp, accessed on 10 June 2022) has been adopted in our experiment to connect external servers for local devices. Two routers have been deployed to achieve NAT penetration: a Vodafone Wi-Fi Hub and a raspberry pi 3B+ with self-compiled OpenWRT firmware. The Frp client was installed in pi-router, and the Frp server was installed in a Google Virtual Private Servers (VPS) with a public IPv4 address. TCPdump tool was also installed in the pi-router to monitor the inbound and outbound traffic of the HAN. The rest of the deployed SH appliances include an SH control platform, a smart lamp, a security camera and an Android smartphone. The SH control platform is made by a Raspberry pi 3B+ with the home assistant firmware; it is responsible to control and monitor the status of all deployed EID in the HAN. The Smart lamp is made by Raspberry pi 3B+ with an LED lights module. The security camera is made by Raspberry pi 3B+ with a camera module. Smart lamp uses an MQTT protocol to receive the command from an SH control platform, and the on/off status of the LED lights is controlled by GPIO pins. The security camera continuously uploads video images to a local server with port 8081. The Android smartphone works as a remote voice control device. A predefined IFTTT applet is associated with the Google Assistant of the smartphone to send the commands to the SH control platform. All SH IoT devices have been assigned with static IP addresses; in this case, the IP addresses of each EID will remain the same after system reboot. The overall architecture of our test-bed SH has been shown in Figure 2.

### 4.2. Data Collection

During data collection, we repeatedly triggered the predefined function feature of the SH IoT device to collect the traffic data. From our observation, generally, the process of turning the smart light on/off finishes in 15 s. Hence, a 15-s time interval has been selected to collect HAN traffic data. The 15-s traffic data will be viewed as a unit and stored separately. The data unit will be used to represent the packets collected in 15 s of time interval from the HAN traffic in the rest of this paper. We have collected 300 data units for each normal scenario, and 600 data units of normal traffic data in total have been collected (https://console.cloud.google.com/storage/browser/ucc_paper_data, accessed on 10 June 2022).

The selected attacks include an SYN flood attack, a DOS attack and an SSH brute force attack. We have launched individual types of selected attacks, respectively, targeting the smart lamp and the SH control platform. We have collected 100 data units of HAN traffic for each type of the attack on the mentioned devices, and 600 units of attack traffic have been collected.

All collected data units are converted to UCC arrays following the process we described in the previous section. In our experiment, each unit of UCC data represents a complete device function activation, which includes full workflow of the control device sending a command and of the end device responding to the command. In a real-world case, the weekly trigger amount of a single device function is generally less than 300 times.Therefore, in this study, we use 300 units of normal UCC array and 300 units of abnormal UCC array to train our classifier. A vector of 791 attributes describes the network behaviours of four devices in then test-bed environment. Smart lamp is the end device, an SH control platform is the control device, and both pi-router and security camera are support evidence devices. Three types of protocols have been considered in this case: TCP, HTTP and MQTT. A detailed explanation of the 791 UCC attributes is listed as follows:

(3) $$\begin{array}{cc} ED_{Total\_Attributes} & =ED_{TCP\_Attributes}+ED_{HTTP\_Attributes}+ED_{MQTT\_Attributes}\\ \; & =(12\times 4)+(17\times 4)+(20\times 4)=196. \end{array}$$

(4) $$\begin{array}{cc} CD_{Total\_Attributes} & =CD_{TCP\_Attributes}+CD_{HTTP\_Attributes}+CD_{MQTT\_Attributes}\\ \; & =(12\times 4)+(17\times 4)+(20\times 4)=196. \end{array}$$

(5) $$\begin{array}{cc} SE1_{Total\_Attributes} & =SE1_{TCP\_Attributes}+SE1_{HTTP\_Attributes}+SE1_{MQTT\_Attributes}\\ \; & =(12\times 4)+(17\times 4)+(20\times 4)=196. \end{array}$$

(6) $$\begin{array}{cc} SE2_{Total\_Attributes} & =SE2_{TCP\_Attributes}+SE2_{HTTP\_Attributes}+SE2_{MQTT\_Attributes}\\ \; & =(12\times 4)+(17\times 4)+(20\times 4)=196. \end{array}$$

(7) $$\begin{array}{cc} UCC\_array,U & =\;ED_{Total\_Attributes}+CD_{Total\_Attributes}+SE1_{Total\_Attributes}\\ \; & +\;SE2_{Total\_Attributes}+Entropy\_Internal_{TCP}+Entropy\_External_{TCP}\\ \; & +\;Entropy\_Internal_{HTTP}+Entropy\_External_{HTTP}\\ \; & +\;Entropy\_Internal_{MQTT}+Entropy\_External_{MQTT}+Label\\ \; & \;=196+196+196+196+1+1+1+1+1+1+1=791. \end{array}$$

### 4.3. Experiments Evaluation

To evaluate our proposed solution, we consider three criteria: (1) different victims with the same triggering scenario; (2) same victim but different involved SH IoT devices; and (3) the amount of data used for training the data models. The measurement used in this paper to indicate include accuracy, precision, recall and F1-score from the confusion matrix. We also consider the time cost to generate the UCC array and the detection model.

(8) $$Accuracy=\frac{TP+TN}{TP+TN+FP+FN}.$$

(9) $$Precision=\frac{TP}{TP+FP}.$$

(10) $$Recall=\frac{TP}{TP+FN}.$$

(11) $$F1\_Score=2\times \frac{Precision\times Recall}{Precision+Recall}.$$

#### 4.3.1. Anomaly Detection under Different Conditions

The first experiment aims to validate that the A-NIDS detection models trained with UCC array data are able to identify attacks under different settings, including:

1. HAN traffic is generated by triggering the smart lamp on/off remotely, meanwhile, the smart lamp is the only victim targeted by the three types of attacks;
2. HAN traffic is generated by triggering the smart lamp on/off remotely, however, the SH control platform is the only victim targeted by the three types of attacks;
3. HAN traffic contains two types of EID network activities: (1) remotely requesting the security camera images; and (2) remotely triggering the smart lamp on/off. The smart lamp is the only victim targeted by the three types of attacks in this case.

**Result Analysis**

Table 1 shows the detection performance of identifying different attacks targeting the smart lamp scenario (we report the average results of 10 rounds of experiments, the same applies in the rest of this paper); 300 units of normal UCC array (triggering smart lamp on/off) and 300 units of abnormal UCC array (different attacks target smart lamp) are used to train each abovementioned ML classifier. Logistic Regression, K-Nearest-Neighbors and SMV perform best in detection, but Logistic Regression and K-Nearest-Neighbors take a longer time to generate the model than others. XGboost takes the longest time to generate a detection model, 3571.6873 s. Naïve Bayes is the most efficient algorithm, which only took 5.127 s. SVM with a linear kernel has the best performance among all models.

Table 2 shows the detection performance of identifying different attacks targeting an SH control platform scenario. The settings are the same as in the previous experiment. The performance of the detection model generated by Logistic Regression, Decision Tree and SVM are the best. Decision Tree is the most efficient algorithm to generate the detection model.

Table 3 shows the detection performance of two types of EID triggered simultaneously. The performance of the detection models generated by Logistic Regression, SVM and Random Forest are the best. SVM with the linear kernel is the most efficient algorithm to generate the detection model.

In conclusion, the detection rates in the above three scenarios indicate that the detection model of A-NIDS generated by UCC data with classification ML algorithms are able to detect the attacks in an SH scenario, and the detection rate of all models has achieved over 99.5%. Moreover, the SVM model is robust for all SH usage cases and is very accurate and efficient in detecting attacks.

#### 4.3.2. Comparisons with Other Under-Sampling Methods

The second experiment aims to show that the A-NIDS detection model trained with UCC data is more efficient and accurate than other methods. We compared the performance of our UCC with other under-sampling methods as a pre-processing approach to generate a detection model in the scenario of a smart lamp being remotely being turned on/off; meanwhile, the lamp is also the only victim targeted by attacks.

**Benchmark Datasets**

The benchmark datasets are also generated from our collected HAN packets data. The k-means has been adopted as the under-sampling method of the benchmark A-NIDS pre-processing approach. The centroid of the k-means cluster of each data file has been used to represent the unit of the HAN network data. Collected packets data will be converted to flow data based on the protocols. Flow data are composed of: (a) protocol-based flow information, (b) file index, and (c) label (normal/abnormal). Protocol-based flow information includes: (1) flow index, (2) flow identity, (3) general flow information, and (4) flow content. General flow information has four attributes, including total number of packets, the average length of the packet, the total length of packets and the average interval time of send/receive packet. TCP flow contains five-tuple attributes as flow identity and six attributes of Flags information as flow content. We also consider the application layer protocols in this case in which HTTP flow contains 3-tuple attributes as flow identity and 11 attributes of response status code as flow content, and MQTT flow contains 3-tuple attributes as flow identity and 14 attributes of command message as flow content. We implement k-means for each protocol group. The selected number of clusters is five, and the k-means cluster centroids of each protocol will then be aggregated with the file index. Each unit of k-means flow data involves 59 attributes. A detailed explanation of the k-means flow data unit is shown below:

(12) $$\begin{array}{cc} TCP\_Flow\_Info & =flow\_index+TCP_{5\_tuple}+TCP_{general\_flow\_info}\\ \; & +\;TCP_{flow\_content}\\ \; & =1+5+4+6=16. \end{array}$$

(13) $$\begin{array}{cc} HTTP\_Flow\_Info & =flow\_index+HTTP_{3\_tuple}+HTTP_{general\_flow\_info}\\ \; & +\;HTTP_{flow\_content}\\ \; & =1+3+4+11=19. \end{array}$$

(14) $$\begin{array}{cc} MQTT\_Flow\_Info & =flow\_index+MQTT_{3\_tuple}+MQTT_{general\_flow\_info}\\ \; & +\;MQTT_{flow\_content}\\ \; & =1+3+4+14=22. \end{array}$$

(15) $$\begin{array}{cc} k-means\_Flow\_Unit,K & =TCP\_Flow\_Info+HTTP\_Flow\_Info\\ \; & +\;MQTT\_Flow\_Info+file\_index+Label\\ \; & =16+19+22+1+1=59. \end{array}$$

**Result Analysis**

The performance of each model using k-means flow data has been shown in Table 4. The time costs of pre-processing by k-means and UCC are shown in Table 5. The time costs for overall processes of k-means A-NIDS and UCC A-NIDS are shown in Table 6.

In general, the detection accuracy of the UCC A-NIDS with the traditional classification ML algorithm is better than the k-means A-NIDS. However, it takes a longer time to generate the detection model. The detection accuracy of the k-means A-NIDS is better than the UCC A-NIDS with ensemble classification ML algorithms. Random Forest took a longer time to generate the detection model. XGboost is more suitable tp use flow data pre-processed by k-means, which requires less time, and the model detection accuracy, recall and f1-score are higher. The data pre-processing is very time-consuming and depends on the volume of datasets. Therefore, when we combine the time of pre-processing and model generation, The UCC A-NIDSs are better than the k-means A-NIDS. In summary, SVM with the linear kernel using UCC data performs best both in detection and time cost.

#### 4.3.3. Minimum Training Requirement of Detection Model

The last experiment aims to identify the minimum training data required for our proposed solution. In the general SH usage case, the frequency of the SH appliance under attack is lower than those when performing predefined tasks normally. Therefore, we randomly selected 25%, 50%, and 75% of 300 units of the attack smart lamp UCC array as the abnormal data, and 300 units of the trigger the smart lamp on/off remotely as the normal UCC array data to generate detection models.

**Result Analysis**

The performance of different detection models is shown in Table 7, Table 8 and Table 9. In general, the detection accuracy and the time cost of model generation are reduced along with the attack datasets reduction. However, SVM with linear kernel can improve the efficiency and remain the same detection accuracy.

### 4.4. Discussion

In summary, we have simulated the most common SH IoT device usage scenario and attacks. Three experiments have illustrated that the detection model generated by the UCC with classical ML classifier has high accuracy in detecting anomalous network activities. The detection results indicate our method is robust to defend network-based attacks in complex IoT-based SH HAN environments. Considering the trade-off between detection performance and resource consumption in SH IoT devices, the SVM with linear kernel function is the most suitable classification algorithm for the analysis engine.

## 5. Conclusions and Future Work

This study proposed a joint training model that combines the UCC method with classification ML algorithms. Instead of using a single IoT device’s network activities to generate a profile, we use SH’s current conditions to profile the overall traffic under a user’s command. The detailed information of packets from both transport and application layer protocols has been used for generating the UCC array and further training the detection model; this enables us to handle the enormous volume of traffic data and reduce the training time for generating the model. Thus, our proposed work can achieve near real-time intrusion detection in the HAN environment. We evaluate the detection performance in a simulated test-bed environment; the results indicate that our solution is superior to others in terms of detection accuracy and efficiency. The detection model generated by the SVM linear kernel with UCC data is robust, efficient and accurate for identify attacks in IoT-based SH HAN environments.

Some limitations will be solved in future work. First, we have not covered different network topologies and protocols in this manuscript. We plan to extend the SH environment to more complex environments incorporating more smart devices. Second, our detection is based on identifying the device-specific communication packets during executing predefined tasks; how to automatically identify the status of the current device by identifying the critical communication packet requires further study. Last, there are peaks and troughs in SH appliance usage scenarios; generating a detection model that simultaneously identifies multiple users’ interactions with different IoT devices needs further exploration.

## Figures and Tables

**Table 1:** Detection performance of identifying different attacks target smart lamp scenario.

	Accuracy	Precision	Recall	F1-Score	Time (s)
Logistic Regression	1	1	1	1	165.5239
Naive Bayes	0.9950	0.9942	0.9970	0.9956	5.1247
Decision Tree	0.9950	0.9973	0.9932	0.9952	26.6726
K-Near-Neighbors	1	1	1	1	375.1953
Support Vector Machine	1	1	1	1	54.2111
Random Forest	0.9983	1	0.9970	0.9985	58.8310
XGboost	0.9983	1	0.9970	0.9984	3571.6873

**Table 2:** Detection performance of identifying different attacks target an SH control platform scenario.

	Accuracy	Precision	Recall	F1-Score	Time (s)
Logistic Regression	1	1	1	1	167.9524
Naive Bayes	0.9967	1	0.9938	0.9969	5.0668
Decision Tree	1	1	1	1	25.9433
K-Near-Neighbors	0.9983	1	0.9970	0.9985	375.6157
Support Vector Machine	1	1	1	1	55.4796
Random Forest	0.9983	1	0.9970	0.9985	52.9531
XGboost	0.9983	1	0.9970	0.9985	3523.0033

**Table 3:** Detection performance of two SH IoT devices triggered simultaneously.

	Accuracy	Precision	Recall	F1-Score	Time (s)
Logistic Regression	1	1	1	1	172.6651
Naive Bayes	0.9967	1	0.9926	0.9962	5.3940
Decision Tree	0.9967	1	0.9935	0.9967	28.1749
K-Near-Neighbors	0.9983	1	0.9966	0.9982	367.3423
Support Vector Machine	1	1	1	1	51.0684
Random Forest	1	1	1	1	55.2915
XGboost	0.9983	1	0.9970	0.9985	3351.2208

**Table 4:** Result of k-means A-NIDS detection.

	Accuracy	Precision	Recall	F1-Score	Time (s)
Logistic Regression	0.9983	0.9980	0.9987	0.9983	74.8578
Naive Bayes	0.5577	0.5322	0.9926	0.6922	2.9718
Decision Tree	0.9297	0.9311	0.9313	0.9293	14.2706
K-Near-Neighbors	0.9923	0.9946	0.9899	0.9922	228.8329
Support Vector Machine	0.9999	0.9987	0.9993	0.9990	29.4634
Random Forest	1	1	1	1	83.6420
XGboost	1	1	1	1	1277.3039

**Table 5:** Pre-processing time of k-means A-NIDS and UCC A-NIDS (in seconds).

	K-Means Flow	K-Means Unit Data	UCC Unit Data
Normal datasets	15.3300	14.5081	17.3926
Attack Datasets	4908.8561	10,481.1166	4953.8402

**Table 6:** Overall processing time of k-means A-NIDS and UCC A-NIDS (in seconds).

	K-Means A-NIDS	UCC A-NIDS
Logistic Regression	15,494.6686	5136.7567
Naive Bayes	15,422.7826	4976.3575
Decision Tree	15,434.0814	4997.9054
K-Near-Neighbors	15,648.6437	5373.1007
Support Vector Machine	15,449.2742	5052.1165
Random Forest	15,503.4528	5056.7364
XGboost	16,697.1147	8569.5927

**Table 7:** Result of 25% attack data.

	Accuracy	Precision	Recall	F1-Score	Time (s)
Logistic Regression	1	1	1	1	94.0099
Naive Bayes	0.9947	0.9931	1	0.9965	3.9892
Decision Tree	0.9866	0.9964	0.9861	0.9912	22.0705
K-Near-Neighbors	1	1	1	1	205.7956
Support Vector Machine	1	1	1	1	5.7967
Random Forest	0.9973	1	0.9966	0.9982	52.2401
XGboost	0.9947	0.9969	0.9966	0.9967	2583.2389

**Table 8:** Result of 50% attack data.

	Accuracy	Precision	Recall	F1-Score	Time (s)
Logistic Regression	1	1	1	1	113.3000
Naive Bayes	0.9977	1	0.9970	0.9985	4.4048
Decision Tree	0.9822	0.9898	0.9829	0.9861	22.2011
K-Near-Neighbors	1	1	1	1	250.4543
Support Vector Machine	1	1	1	1	15.1883
Random Forest	0.9978	1	0.9970	0.9985	56.8939
XGboost	0.9956	0.9968	0.9970	0.9968	3028.6145

**Table 9:** Result of 75% attack data.

	Accuracy	Precision	Recall	F1-Score	Time (s)
Logistic Regression	1	1	1	1	135.0208
Naive Bayes	0.9962	0.9962	0.9967	0.9964	4.6284
Decision Tree	0.9904	0.9908	0.99323	0.9920	24.2143
K-Near-Neighbors	1	1	1	1	300.4641
Support Vector Machine	1	1	1	1	32.6009
Random Forest	0.9981	1	0.9967	0.9983	55.1996
XGboost	0.9962	1	0.9935	0.9967	3296.4205

## Data Availability

Not applicable.

## Abbreviations

The following abbreviations are used in this manuscript:

A-NIDS	Anomaly-based Network Intrusion Detection System
CD	Control Device
EID	End Smart Home IoT Device
HAN	Home Area Network
HG	Home gateway
IoT	Internet of Things
ML	Mechanical Learning
NIDS	Network-based Intrusion Detection System
OS	Operating Systems
SH	Smart Home
S-NIDS	Signature-based Network Intrusion Detection System
UCC	User Command Chain
